# Polymicrobial Prosthetic Joint Infections: Unraveling Risk Factors and Outcomes in a Single-Center Study

**DOI:** 10.3390/microorganisms13071679

**Published:** 2025-07-16

**Authors:** Álvaro Auñón, Ignacio Ortiz, Salvador Peñarrubia, Carmen Álvaro, Estíbaliz Torrecilla-Sádaba, Joaquin Garcia-Cañete, Jaime Esteban

**Affiliations:** 1 Department of Orthopaedic Surgery, Fundación Jiménez Díaz University Hospital, 28040 Madrid, Spain; alvaro.aunon@fjd.es (Á.A.); ignacio.ortizm@quironsalud.es (I.O.); salvador.penarrubia@quironsalud.es (S.P.); 2 CIBERINFEC-CIBER de Enfermedades Infecciosas, 28029 Madrid, Spain; 3 Department of Internal Medicine, Fundación Jiménez Díaz University Hospital, 28040 Madrid, Spain; carmen.alvarov@quironsalud.es (C.Á.); jgarciac@fjd.es (J.G.-C.); 4 Department of Clinical Microbiology, IIS-Fundacion Jimenez Diaz, Universidad Autónoma de Madrid (UAM), 28040 Madrid, Spain; estibaliz.torrecilla@quironsalud.es

**Keywords:** periprosthetic joint infection (PJI), polymicrobial, monomicrobial, risk factor, coagulase-negative staphylococci (CoNS), gram-negative bacilli, *S. aureus*

## Abstract

Periprosthetic joint infection (PJI) is a serious complication after joint arthroplasty, with polymicrobial PJIs representing a distinct subset associated with worse outcomes. This study aims to characterize the risk factors, microbiological profiles, and clinical outcomes of polymicrobial PJIs in a single tertiary care center. A retrospective analysis was conducted on 499 patients diagnosed with PJI between 2010 and 2023. Polymicrobial infection was defined by isolation of ≥2 distinct pathogens from intraoperative samples. Demographic, microbiological, and clinical data were analyzed. Treatment success was defined as infection eradication without recurrence or chronic suppressive therapy. Polymicrobial PJIs accounted for 18.2% of cases. Patients with polymicrobial infections had higher rates of obesity, insulin-dependent diabetes, and higher Charlson comorbidity scores. Coagulase-negative staphylococci and gram-negative bacilli were more frequently isolated in polymicrobial infections, while *S. aureus* predominated in monomicrobial cases. Treatment success rates were significantly lower in polymicrobial infections, both in acute (61.5% vs. 94.5%, *p* = 0.003) and chronic settings (51.3% vs. 75.3%, *p* = 0.02). Polymicrobial PJIs are associated with distinct microbiological patterns, increased comorbidity burden, and significantly worse clinical outcomes. Recognition of specific risk factors and pathogen profiles is essential to optimize management strategies for this complex condition.

## 1. Introduction

Total joint arthroplasty (TJA) is one of the most commonly performed elective orthopedic procedures worldwide and has become a cornerstone in the management of end-stage degenerative joint disease. It significantly enhances patients’ quality of life by effectively relieving chronic pain, restoring mobility, and improving overall joint function and independence in daily activities. Over recent decades, continuous improvements in surgical techniques, perioperative protocols, prosthetic materials, anesthesia, rehabilitation strategies, and infection prevention measures have contributed to a substantial reduction in complications. Nevertheless, periprosthetic joint infection (PJI) remains one of the most devastating and feared postoperative complications, with an incidence ranging from approximately 1.5% to 2.5% of cases [1]. While this percentage may seem relatively low, the absolute number of cases continues to rise due to the increasing global volume of TJAs performed each year, particularly in aging populations with multiple comorbidities.

PJIs exert a considerable burden on patients and healthcare systems alike. These infections often necessitate prolonged hospitalizations, multiple surgical interventions—including complex revision procedures—and long-term administration of systemic antibiotic therapy, often with agents that carry significant side effects or require parenteral delivery [2]. In addition to the direct clinical consequences, PJIs are also associated with substantial psychological distress, reduced quality of life, loss of independence, increased healthcare costs, and a higher risk of morbidity and mortality [3,4,5]. The emotional toll on patients and their families is often underestimated, further complicating recovery and rehabilitation. Although monomicrobial PJIs have been thoroughly studied and are relatively well-characterized in clinical guidelines, polymicrobial PJIs represent a more complex and less understood clinical entity.

Polymicrobial PJIs, defined by the isolation of two or more distinct microorganisms from the infected joint, are estimated to account for approximately 10–15% of all PJI cases [1,5,6]. These infections typically involve a combination of aerobic and anaerobic organisms, with frequent identification of *Staphylococcus aureus*, coagulase-negative staphylococci (CoNS), *Enterococcus* spp., gram-negative bacilli, and obligate anaerobes [1,7]. The presence of multiple species creates a complex microbial ecosystem that enhances bacterial survival through interspecies cooperation, particularly within the protective environment of the biofilm. Moreover, these interactions can lead to increased production of virulence factors and metabolic byproducts that further impair host response. Biofilm formation remains the central pathogenic mechanism in PJIs, and in polymicrobial settings, the synergistic behavior among species embedded within the matrix contributes to increased resistance to both antimicrobial agents and host immune defense [1,8]. This heightened resilience complicates eradication efforts and often necessitates more aggressive surgical and pharmacological strategies.

Several host and surgical factors have been identified as predisposing elements for polymicrobial infection. These include prior revision surgeries, the presence of sinus tracts or chronic wound drainage, a high body mass index, and other comorbid conditions such as diabetes or immunosuppression [5,9]. Patients with these risk factors may have repeated exposures to healthcare environments, surgical interventions, or broad-spectrum antibiotics, increasing the likelihood of colonization and subsequent infection by multiple organisms. Additional contributors include prolonged postoperative wound healing, repeated diagnostic aspirations, and prior episodes of infection or inflammation at the surgical site. The unique microbiological profile of polymicrobial PJIs often necessitates broader-spectrum empirical antibiotic coverage, followed by combination regimens that simultaneously target gram-positive cocci, gram-negative bacilli, and anaerobes. This approach, although often necessary, carries a higher risk of antimicrobial toxicity and may contribute to the development of multidrug resistance [7,10]. Close multidisciplinary collaboration is essential to optimize treatment outcomes while minimizing complications.

Managing polymicrobial PJIs remains a significant clinical challenge. Available evidence consistently demonstrates that these infections are associated with higher rates of treatment failure, reinfection, and the need for salvage procedures compared to monomicrobial cases. Debridement, antibiotics, and implant retention (DAIR)—a widely used option in acute PJI—shows markedly lower success rates in polymicrobial infections, with some studies reporting eradication in fewer than 60% of cases [11]. These poorer outcomes may be attributed to the increased microbial diversity, biofilm complexity, and delayed diagnosis frequently observed in polymicrobial presentations. In contrast, two-stage exchange arthroplasty, although more invasive and resource-intensive, appears to yield better outcomes, particularly in chronic or recurrent infections. Furthermore, recent studies have drawn attention to the increased risk of severe complications such as limb amputation, joint arthrodesis, prolonged disability, extended rehabilitation, and infection-related mortality among patients with polymicrobial PJIs [4,5]. Early identification and individualized treatment strategies are therefore essential to improve prognosis.

These findings collectively highlight the pressing need for more accurate diagnostic strategies and individualized treatment approaches. Traditional culture-based diagnostics may fail to identify all involved organisms, especially in the context of prior antibiotic exposure or slow-growing pathogens. Additionally, polymicrobial infections may exhibit selective overgrowth, masking fewer dominant species. Newer molecular techniques such as classic 16S polymerase chain reaction (PCR), multiplex PCR assays, and next-generation sequencing (NGS) hold promise for improving microbial detection, but their clinical adoption remains limited due to cost, accessibility, and interpretative challenges. Moreover, standardization of these technologies, regulatory approval, and integration into clinical workflows are necessary before they can be routinely implemented.

Despite their clear clinical relevance, polymicrobial PJIs continue to be underrepresented in current management guidelines, which largely focus on monomicrobial infections. As a result, clinicians are often left to extrapolate treatment strategies based on limited evidence, case series, or expert opinion. There is a growing recognition that polymicrobial infections require a distinct diagnostic and therapeutic framework that accounts for their microbiological complexity, resistance patterns, variable host responses, and host-related risk factors. A more nuanced understanding is essential for guiding individualized care and optimizing outcomes.

This study aims to provide a comprehensive overview of polymicrobial PJIs, examining their microbiological spectrum, associated risk factors, clinical outcomes, and response to various treatment modalities. By better understanding the specific characteristics of these infections, we hope to inform clinical practice and contribute to the development of more effective, evidence-based protocols that improve patient outcomes in this increasingly recognized and challenging condition. Additionally, we aim to identify current gaps in knowledge and propose directions for future research to address this evolving clinical issue.

## 2. Materials and Methods

A retrospective observational study was conducted in a third-level university hospital, including all patients diagnosed with periprosthetic joint infection (PJI) at our center between January 2010 and December 2023. Data were collected from electronic medical records and analyzed in compliance with institutional ethical guidelines. The study was approved by the local ethics committee. Demographic variables such as age, gender, American Society of Anesthesiologists (ASA) classification, and Charlson Comorbidity Index (CCI) were recorded. Additional patient-related factors included body mass index (BMI), smoking status, alcohol consumption, and presence of diabetes mellitus or immunosuppression. Regarding surgical data, the type of joint (hip or knee), nature of the index surgery (primary, revision, or fracture-related), and subsequent surgical interventions were documented in detail. The interval between index arthroplasty and infection onset was also registered to categorize infections as acute or chronic. All patients underwent a standardized preoperative diagnostic protocol. This included routine blood tests (white blood cell count, erythrocyte sedimentation rate, and C-reactive protein), radiographic evaluation, and, when feasible, joint aspiration for microbiological and cytological analysis. Synovial fluid was analyzed for leukocyte count and percentage of polymorphonuclear cells. Intraoperative tissue and fluid samples were systematically collected and sent for microbiological analysis. Samples were processed following the protocol established by our institution and described in a previous publication by our research group [12]. Briefly, tissues were processed by grinding, and implants were sonicated in sterile plastic containers after adding 100 mL of pH 6.8 phosphate buffer in a low power ultrasound sonicator (J.P.Selecta, SA., Barcelona, Spain). After sonication, the sonicate fluid was centrifuged at 3000× *g* for 20 min, and the sediment was resuspended in 10 mL of the same buffer. A 10 µL amount of the concentrate was inoculated in plates with the following media: tryptic soy 5% sheep blood agar (TSS), chocolate agar (CH), and McConkey (McC) agar for aerobic culture, and Schaedler 5% sheep blood agar (SCS) for anaerobic culture (all media from Biomérieux, Marcy l’Etoile, France). The media were incubated at 37 °C in 5% CO_2_ atmosphere (TSS and CH), normal atmosphere (McC), and anaerobic atmosphere (SCS). All culture media were incubated for at least 14 days, except for McC experiments, which were incubated for 24 h. Microorganisms were identified using matrix-assisted laser desorption/ionization time-of-flight (MALDI-ToF) mass spectrometry. Since 2014, microorganisms have been identified using this technology. Prior to this, identification was based on commercial biochemical tests, following the diagnostic criteria accepted at the time. Antibiotic susceptibility testing was performed using the broth microdilution method and interpreted according to the European Committee on Antimicrobial Susceptibility Testing (EUCAST) guidelines. Microorganisms were classified according to resistance profiles: no resistance, gram-positive resistance to rifampin, gram-negative resistance to quinolones, and multidrug resistance (MDR) as defined by Magiorakos et al. [13]. The antimicrobial susceptibility testing of the organisms was performed using an automated system (VITEK^®^ 2, Biomérieux) and, in some cases, Etest and disc–plate assays. The tested antibiotics were those included in the VITEK^®^ cards and, for manual methods, those recommended for specific microorganisms. We considered rifampin and quinolones as the key antibiotics for the treatment of gram-positive (especially staphylococcal) and gram-negative organisms, respectively, as previous articles have demonstrated in the literature [13].

In cases of polymicrobial infection, resistance was assessed individually for each isolate and collectively to guide antimicrobial treatment decisions. The diagnosis of PJI was established according to the 2018 ICM criteria [14] following preoperative tests such as white blood cell counts, erythrocyte sedimentation rate, C-reactive protein measurements, and, when feasible, joint aspiration and microbiological cultures from 5 to 7 samples. The date of diagnosis was defined as the date of the initial surgical intervention when positive cultures were obtained. Cases diagnosed prior to 2018 were retrospectively classified according to the 2018 ICM criteria.

The multidisciplinary infection unit, composed of orthopedic surgeons, infectious disease specialists, and clinical microbiologists, met weekly to evaluate each case. Treatment strategies were individualized based on patient comorbidities, infection severity, and microbiological findings. Patients underwent one of the following surgical strategies: debridement, antibiotics, and implant retention (DAIR); one-stage revision arthroplasty; or two-stage revision arthroplasty. Salvage procedures such as arthrodesis or amputation were considered in cases of treatment failure or recurrent infection. The decision-making process was guided by institutional protocols but allowed for clinical discretion.

Antimicrobial therapy was tailored to culture results and administered according to international guidelines. Intravenous antibiotics were typically initiated immediately postoperatively and continued for a minimum of 2 to 6 weeks, followed by oral therapy when indicated. Antimicrobial duration and changes were recorded in the patient file.

A minimum follow-up period of 12 months from the final surgical intervention was required for inclusion. Patients with less than 12 months of follow-up or those lost to follow-up were excluded from the final analysis. Loss to follow-up was defined as the absence of any clinical visit or laboratory evaluation during the 12-month window after definitive treatment. The primary outcome was treatment success, defined as eradication of infection with no clinical signs, no need for suppressive antibiotics, and no additional surgery, according to MusculoSkeletal infection Society (MSIS) [15]. Treatment failure included persistent or recurrent infection, the need for further surgical procedures, or infection-related mortality.

## 3. Results

A total of 536 patients diagnosed with periprosthetic joint infection (PJI) were identified and initially considered for inclusion in the present study. After applying exclusion criteria (less than 12 months of follow-up or loss to follow-up), 499 patients were evaluated. Among them, 91 cases (18.2%) were classified as polymicrobial infections, defined by the isolation of two or more distinct microorganisms from intraoperative tissue or fluid cultures obtained during revision surgery. These cultures were processed using standardized microbiological protocols to ensure the accuracy and reproducibility of results. The remaining 408 cases were classified as monomicrobial and served as the comparison group for clinical and microbiological outcome analysis.

The mean age of the study cohort was 74.5 years. The sex distribution showed a modest predominance of female patients (56.1%). The hip joint was the most frequently involved site, accounting for 57.7% of PJIs, while the knee joint was affected in 42.3% of cases. 

When considering the type of implant involved, 53% of infections occurred in primary prosthetic implants, 30.9% in revision prostheses, and 16.1% were associated with fracture-related prosthetic implants, including those placed for trauma or non-elective indications. A total of 368 patients (73.7%) were diagnosed with acute infection, while 131 (26.3%) were diagnosed with chronic infection, based on established clinical and microbiological criteria.

From a surgical risk perspective, the mean ASA Score was 2.48; furthermore, the mean Charlson Comorbidity Index (CCI) was 4.61, indicating a moderately comorbid population. Assessment of body mass index (BMI) showed a broad distribution across the cohort: 6.8% of patients had a BMI < 19 (underweight), 36.7% fell within the normal range (20–25), 37.3% were classified as overweight (25–30), and 19.2% met the criteria for obesity (BMI > 30). Demographic data and comorbidity profiles are shown in Table 1 for further reference.

Regarding pathogens, coagulase-negative staphylococci (CoNS) were the most frequently isolated microorganisms, accounting for 38.4% of all culture-positive cases. *Staphylococcus aureus* was identified in 32.9% of cases. Gram-negative bacilli were isolated in 21.8% of patients, with *Escherichia coli*, *Klebsiella* spp., and *Pseudomonas aeruginosa* among the most common. The full spectrum of isolated pathogens, including their frequency and distribution across mono- and polymicrobial infections, is detailed in Table 2.

As for related risk factors, 11.85% of the patients had insulin-dependent diabetes mellitus. Additionally, 25.3% of patients were current smokers. Furthermore, 13.4% reported chronic alcohol use. In terms of infection onset, 41.7% of PJIs were classified as acute postoperative infection. The majority of cases (51.6%) were identified as chronic infections. A smaller proportion of the patients (6.8%) were deemed hematogenous in origin. Additionally, antimicrobial resistance was identified in 36.1% of cases.

When comparing both groups, the overall treatment success rate was notably lower among patients diagnosed with polymicrobial infections compared to those with monomicrobial infections, a difference that reached statistical significance (*p* = 0.002). Importantly, no infection-related deaths were reported in either group during the follow-up period. When stratified by type of infection, the differences in treatment success remained statistically significant. In cases of acute infection, success rates were significantly higher in the monomicrobial group (94.5%) compared to the polymicrobial group (61.5%, *p* = 0.003). Similarly, in chronic infections, the success rate was 75.3% in monomicrobial cases versus 51.3% in polymicrobial infections (*p* = 0.02).

Regarding the isolated pathogens, *Staphylococcus aureus* was the most frequently identified microorganism in the monomicrobial group, accounting for 36.3% of isolates. This was followed by coagulase-negative staphylococci (CoNS) at 22.5% and gram-negative bacilli at 17.4%. 

In contrast, the microbiological profile of the polymicrobial group revealed a different pattern: CoNS were the most commonly isolated organisms, present in 57.1% of cases, followed closely by gram-negative bacilli at 53.8% and *S. aureus* at a lower frequency of 17.5%. These findings suggest a distinct microbial landscape in polymicrobial infections. A statistically significant association was observed between *S. aureus* and monomicrobial infections (*p* = 0.04), while both CoNS (*p* = 0.07, near significance) and gram-negative bacilli (*p* = 0.0003) were significantly associated with polymicrobial infections. Furthermore, a significant difference in the patterns of antimicrobial resistance was noted between the two groups (*p* = 0.017), with a higher prevalence of resistant organisms observed in the polymicrobial group. The most frequent polymicrobial combinations were CoNS + *E. coli* (7%), followed by CoNS and *Enterococcus* (6%) and *Enterococcus* spp. and GNB (5%). The data regarding microbial combinations are detailed in Table 3.

In terms of comorbidities, patients with polymicrobial infections presented with a significantly higher body mass index (BMI > 30), which was found to be statistically significant (*p* = 0.04). Additionally, the prevalence of diabetes mellitus was significantly greater in the polymicrobial group (*p* = 0.009). The Charlson Comorbidity Index (CCI), which provides a composite measure of patient health status and predicted mortality, was also significantly higher in this group (mean score 6.1 vs. 3.6; *p* = 0.009), indicating a more comorbid and clinically vulnerable population. Data pertaining to the main risk factors are detailed in Table 4.

## 4. Discussion

Periprosthetic joint infections (PJIs) place a heavy burden on healthcare systems due to extended hospital stays, complex revision surgeries, and prolonged antibiotic treatments. Despite their relatively low incidence compared to other arthroplasty complications, PJIs remain one of the most devastating events following joint replacement, with significant consequences for patient mobility, function, and quality of life. These infections often necessitate multidisciplinary care, repeated interventions, and long-term follow-up, contributing to increased healthcare expenditure and resource utilization. Moreover, the psychological impact on patients, including anxiety, depression, and social isolation, is frequently underrecognized but plays a crucial role in the overall recovery and long-term outcomes.

According to current evidence, the incidence of polymicrobial periprosthetic joint infections varies widely, ranging from 6% to 37% of cases [16,17,18] depending on diagnostic criteria, patient population, and surgical context. In our cohort, 18.2% of PJIs were polymicrobial, falling within the reported range and confirming their non-negligible presence in clinical practice.

While monomicrobial PJIs are well understood and extensively studied, polymicrobial infections are comparatively underrepresented in the literature, despite growing evidence that they are associated with worse clinical outcomes [2,5], as well as an elevated treatment cost [17]. Our findings reinforce this trend, showing significantly lower treatment success rates in patients with polymicrobial infections—both in acute and chronic settings—compared to their monomicrobial counterparts. These results are consistent with previous studies by Kavolus et al. [2] and Wimmer et al. [4], which reported similar outcome disparities, particularly after DAIR procedures.

The complex microbial environment, increased biofilm resilience, and broader antimicrobial resistance profiles commonly found in polymicrobial PJIs likely contribute to the reduced efficacy of standard surgical and antibiotic interventions. These infections often involve synergistic microbial interactions that enhance virulence and impair host immune responses, creating a more hostile and treatment-resistant environment. Additionally, the diagnostic challenges posed by mixed infections can lead to delays in appropriate therapy, further compromising outcomes. As a result, treatment strategies must be adapted to address the multifaceted nature of these infections, often requiring prolonged combination antibiotic regimens, more extensive surgical debridement, and close interdisciplinary collaboration.

Several mechanisms may contribute to these poorer outcomes. The presence of multiple bacterial species within a biofilm creates a complex microenvironment that enhances microbial survival and antibiotic resistance. Synergistic interactions between organisms inside the biofilm can increase tolerance to host immune responses and antimicrobial agents. Moreover, biofilms impair antibiotic penetration and facilitate horizontal gene transfer, further promoting resistance [19]. This was reflected in our data, where antibiotic resistance—especially to quinolones and rifampin—was significantly more common in polymicrobial PJIs.

The specific microbiological profile of polymicrobial PJIs differs notably from monomicrobial cases. Our study confirms the overrepresentation of CoNS and gram-negative bacilli in polymicrobial infections, whereas *Staphylococcus aureus* was more common in monomicrobial PJIs. These results align with findings by Flurin et al. [1], who also observed a strong association between *S. epidermidis* and polymicrobial infection. The increased prevalence of CoNS may be explained by their capacity to form robust biofilms and survive in suboptimal conditions, such as those created by surgical trauma, hematoma, or necrotic tissue. Furthermore, CoNS often act as “foundation bacteria” that facilitate subsequent colonization by more aggressive organisms, such as gram-negative rods or enterococci. This microbial cooperation not only increases ecological stability within the infection site but also enhances resistance to host defenses and antimicrobial agents, complicating eradication and contributing to the chronicity and recurrence of infection [1,20]. Other works [5,17] have also identified a significant association between the presence of gram-negative bacilli and the occurrence of polymicrobial periprosthetic joint infections (PJIs). These findings suggest that gram-negative organisms may play a pivotal role in the pathogenesis of complex infections, often coexisting with other microbial species and contributing to increased biofilm formation, antimicrobial resistance, and therapeutic challenges. Regarding *Enterococcus* spp., it is frequently isolated in polymicrobial infections because of its relatively low intrinsic virulence, allowing it to coexist with other bacteria without outcompeting them. Additionally, its ability to form biofilms and survive in harsh environments, such as wounds or prosthetic materials, supports its persistence alongside more aggressive pathogens. Clinical studies show that in prosthetic joint infections (PJIs), enterococci are found in mixed cultures in over 50% of cases, particularly with organisms like coagulase-negative staphylococci and gram-negative bacilli, suggesting a synergistic role in chronic infections where multiple pathogens can colonize and evade host defenses together [21,22].

Interestingly, our cohort also demonstrated an association between polymicrobial PJIs and certain host-related risk factors. Obesity, diabetes mellitus, and a higher comorbidity burden (as indicated by elevated Charlson scores) were all significantly more prevalent in the polymicrobial group. These findings echo those reported in previous works [23,24], highlighting the importance of metabolic and systemic factors in infection susceptibility. Obese patients, for example, often experience impaired wound healing and altered antibiotic pharmacokinetics, both of which can contribute to infection chronicity and microbial complexity.

From a therapeutic standpoint, polymicrobial PJIs present formidable challenges because of the need to adapt to the different resistance patterns of the different organisms that appear in the cultures. Our results show that DAIR, although commonly attempted in acute cases, had significantly lower success rates in polymicrobial infections. This supports the idea that DAIR may not be sufficient in cases involving biofilm-forming, multidrug-resistant organisms. In contrast, two-stage revision arthroplasty appeared to offer more reliable outcomes, even though success rates remained suboptimal. These findings are in line with those of Marculescu et al. [3], who also noted that while two-stage revisions could improve eradication rates, patients with polymicrobial infections still had higher risks of recurrence and functional compromise.

Additionally, the prolonged hospital stays and more frequent need for broad-spectrum or combination antibiotic therapy observed in polymicrobial PJIs reflect their clinical complexity. This fact increases its importance where an oral treatment is designed for each case. In this setting, the need for different antibiotic combinations can increase their side effects, together with different interactions between them and with other treatments that are common among these patients because of their concomitant diseases. Beyond the direct patient impact, this also has important implications for antimicrobial stewardship and healthcare resource allocation. The need for extended intravenous therapy, the use of last-resort antibiotics, and higher rates of reoperation all contribute to the overall burden. Moreover, the detection of resistant organisms inside the polymicrobial infection can lead to the selection of broad-spectrum antibiotics, which can be a potential problem for the development of new resistances inside the biofilm because of the complex interactions that appear between the different species.

It is also worth noting that traditional culture techniques may underestimate the true prevalence of polymicrobial infections, especially in patients previously treated with antibiotics. The emergence of sonication in the last few years of the first decade of this century has increased the rate of polymicrobial infection identification because of the increased sensitivity and specificity of this method [12]. Other improvements in culture techniques (prolonged incubation and use of liquid media in some cases) have added more sensitivity to the detection of these infections in recent years. Emerging molecular diagnostic methods such as NGS and multiplex PCR offer improved sensitivity and may reveal additional organisms not detected in conventional culture [25]. However, these tools are not yet widely implemented and raise issues of interpretation, particularly in distinguishing pathogens from contaminants or colonizers; however, increasing knowledge regarding these techniques and diseases make them an option for the high-specificity diagnosis of implant-associated infections [26].

Our study has several limitations. It is a single-center, retrospective analysis, which may introduce selection bias and limit generalizability. Additionally, while we applied strict diagnostic criteria and standardized microbiological techniques, variations in culture sensitivity and prior antibiotic use may still affect the results. Despite these limitations, the size of our cohort and the comprehensive microbiological analysis provide valuable insight into the characteristics and outcomes of polymicrobial PJIs. Moreover, all these patients were evaluated and followed by a multidisciplinary team that included trauma surgeons, infectious disease specialists, and microbiologists, increasing the probability of adequate diagnosis and evaluation of the patients.

## 5. Conclusions

Polymicrobial periprosthetic joint infections (PJIs) represent a particularly complex and clinically relevant subgroup that is associated with increased comorbidity, higher rates of antimicrobial resistance, and poorer outcomes compared to monomicrobial cases. The management of PJIs is often complicated by delayed diagnosis, the presence of biofilm-forming organisms, and the need for broad-spectrum or combined antibiotic therapies. Identifying predisposing factors—such as obesity, diabetes, and history of previous surgeries—remains essential for early intervention and tailored treatment planning.

Moving forward, optimizing antibiotic regimens—especially for outpatient care—requires careful consideration of drug interactions, tolerability, and the specific challenges posed by polymicrobial flora. In parallel, research into new anti-biofilm strategies and personalized treatment approaches based on host and pathogen characteristics is urgently needed. Multicenter prospective studies will be key to refining diagnostic criteria and treatment algorithms, ultimately improving outcomes in this high-risk patient population.

## Figures and Tables

**Table 1:** Demographic data.

	Monomicrobial (n = 408)	Polymicrobial (n = 99)
Age (Years)	73.6 (34–97)	77.8 (35–98)
Sex (Male/Female)	223/185	33/58
Joint (Hip/Knee)	239/169	52/39
ASA	2.52	2.34
BMI ≥ 30 (%)	17.4%	29.6%
Charlson Score	3.6	6.1

ASA: American Society of Anesthesiologists classification; BMI: Body Mass Index.

**Table 2:** Microbiological isolates and % of the defined antibiotic resistance by group.

	Monomicrobial (n = 408)	Polymicrobial (n = 99)
*S. aureus*	36.3%	17.6%
CoNS	22.5%	67%
*Streptococcus* spp.	3.7%	9.9%
*Enterococcus* spp.	3.4%	28.6%
*E. coli*	3.9%	30.8%
Gram-negative bacilli	17.4%	41.7%
Atypical bacteria	6.9%	15.4%
Gram-positive bacilli	3.9%	17.6%
Fungi	2%	4.4%
Antibiotic Resistance	32.6%	46.1%

CoNS: Coagulase-Negative *Staphylococcus.*

**Table 3:** Main microbiological combinations isolated from the polymicrobial group.

Microorganism Combinations	N (% of Total)
CoNS *+ E. coli*	7 (7.7%)
CoNS + *Enterococcus* spp.	6 (6.6%)
*Enterococcus* spp. + GNB	5 (5.5%)
GNB + unusual bacteria	4 (4.4%)
CoNS + unusual bacteria	4 (4.4%)
*E. coli + Enterococcus* spp.	4 (4.4%)
CoNS + *S. aureus*	4 (4.4%)
*E. coli* + other GNB	3 (3.3%)
CoNS + GNB + *Enterococcus* spp.	3 (3.3%)

CoNS: Coagulase-Negative *Staphylococcus*; GNB: Gram-Negative Bacilli.

**Table 4:** Main risk factors.

Main Risk Factors	Monomicrobial (%)	Polymicrobial (%)
Diabetes mellitus	41(10%)	18 (19.8%)
COPD	30 (7.3%)	4 (4.4%)
Alcoholism	56 (13.7%)	11(12.1%)
Immunosuppression	18 (4.4%)	3 (3.3%)
Cirrhosis	38 (9.3%)	10 (11%)
CKD	55 (13.5%)	11 (1.1%)

COPD: Chronic Obstructive Pulmonary Disease; CKD: Chronic Kidney Disease.

## Data Availability

The original contributions presented in this study are included in the article. Further inquiries can be directed to the corresponding author.

## Abbreviations

The following abbreviations are used in this manuscript:

PJI	Periprosthetic joint infection
DAIR	Debridement, antibiotics, and implant retention
ASA	American Society of Anesthesiologists
MSIS	Musculo Skeletal infection Society
CCI	Charlson Comorbidity Index
BMI	Body mass index
MALDI-ToF	Matrix-assisted laser desorption/ionization time-of-flight
EUCAST	European Committee on Antimicrobial Susceptibility Testing
CoNS	Coagulase-negative staphylococci
COPD	Chronic obstructive pulmonary disease
CKD	Chronic kidney disease
